# Occurrence and mechanisms of tigecycline resistance in carbapenem- and colistin-resistant *Klebsiella pneumoniae* in Thailand

**DOI:** 10.1038/s41598-024-55705-2

**Published:** 2024-03-03

**Authors:** Nachat Chirabhundhu, Sirirat Luk-In, Thanawat Phuadraksa, Sineewanlaya Wichit, Tanittha Chatsuwan, Dhammika Leshan Wannigama, Sakda Yainoy

**Affiliations:** 1 Department of Clinical Microbiology and Applied Technology, Faculty of Medical Technology, Mahidol University, Nakhon Pathom, Thailand; 2 Department of Microbiology, Faculty of Medicine, Chulalongkorn University, King Chulalongkorn Memorial Hospital, Thai Red Cross Society, Bangkok, Thailand; 3 Center of Excellence in Antimicrobial Resistance and Stewardship, Faculty of Medicine, Chulalongkorn University, Bangkok, Thailand; 4 Department of Infectious Diseases and Infection Control, Yamagata Prefectural Central Hospital, Yamagata, Japan; 5 School of Medicine, Faculty of Health and Medical Sciences, The University of Western Australia, Nedlands, WA Australia; 6 Biofilms and Antimicrobial Resistance Consortium of ODA Receiving Countries, The University of Sheffield, Sheffield, UK; 7 Pathogen Hunter’s Research Collaborative Team, Department of Infectious Diseases and Infection Control, Yamagata Prefectural Central Hospital, Yamagata, Japan

**Keywords:** Infectious diseases, Antimicrobials, Bacteria, Bacteriology, Clinical microbiology, Microbial genetics

## Abstract

Tigecycline has been regarded as one of the most important last-resort antibiotics for the treatment of infections caused by extensively drug-resistant (XDR) bacteria, particularly carbapenem- and colistin-resistant *Klebsiella pneumoniae* (C-C-RKP). However, reports on tigecycline resistance have been growing. Overall, ~ 4000 *K. pneumoniae* clinical isolates were collected over a five-year period (2017–2021), in which 240 isolates of C-C-RKP were investigated. Most of these isolates (91.7%) were resistant to tigecycline. Notably, a high-risk clone of ST16 was predominantly identified, which was associated with the co-harboring of *bla*_NDM-1_ and *bla*_OXA-232_ genes. Their major mechanism of tigecycline resistance was the overexpression of efflux pump *acrB* gene and its regulator RamA, which was caused by mutations in RamR (M184V, Y59C, I141T, A28T, C99/C100 insertion), in RamR binding site (PI) of *ramA* gene (C139T), in MarR (S82G), and/or in AcrR (L154R, R13Q). Interestingly, four isolates of ST147 carried the mutated *tet*(A) efflux pump gene. To our knowledge, this is the first report on the prevalence and mechanisms of tigecycline resistance in C-C-RKP isolated from Thailand. The high incidence of tigecycline resistance observed among C-C-RKP in this study reflects an ongoing evolution of XDR bacteria against the last-resort antibiotics, which demands urgent action.

## Introduction

Antimicrobial resistance (AMR) is an inevitable evolutionary process conducted by organisms through genetic mutation to survive in lethal selective pressure, particularly antimicrobial agents. It has been declared by the World Health Organization that AMR is one of the top ten global public health threats confronting humanity^1^. According to the comprehensive statistical analysis of data globally collected in 2019, AMR was estimated to be associated with more than 4.9 million deaths^2^. Unless the action is taken, the burden of AMR is predicted to cause 100 trillion USD cumulative global economic cost per year by 2050^3^. One of the most problematic AMR bacteria is carbapenem-resistant Enterobacterales (CRE), especially *Klebsiella pneumoniae*, which has been reported with the highest rate of carbapenem resistance among the members of the Order^2^. *K. pneumoniae* is a natural inhabitant of gastrointestinal tract of healthy humans and animals. It is a potential community-acquired pathogen and a common nosocomial pathogen causing urinary tract infection, pneumonia, meningitis, and sepsis, which account for about one-third of all gram-negative infections^4^. As the treatment continues, the global prevalence of antimicrobial resistance in *K. pneumoniae* is progressively rising^5^. The emergence of carbapenem-resistant *K. pneumoniae* (CRKP) has given the worst challenge to global public health since it has seriously restricted the available treatment options to specific antibiotics such as colistin, tigecycline, aminoglycosides, and fosfomycin^6^. Furthermore, because of colistin usage, colistin resistance has also emerged among CRKP, which resulted in an extremely drug-resistant phenotype. The occurrence of carbapenem- and colistin-resistant *K. pneumoniae* (C-C-RKP) intensified the risk posed by AMR, as colistin is one of the limited remaining treatment options. To deal with such extreme resistant phenotype, the remaining last-resort antibiotic, tigecycline, has been prescribed^6^. Tigecycline is a minocycline-derivative semisynthetic glycylcycline class antibiotic^7^. It has a bacteriostatic activity and similar mechanism of action to other tetracyclines by acting as a bacterial protein translation inhibitor interrupting an elongation of the peptide chain via reversible binding to 30s subunit of bacterial ribosome, which results in prevention of amino acid residue incorporation, lack of peptide chain formation, and consequently inhibit bacterial growth^8^. Moreover, the unique structure of tigecycline results in its broad spectrum of activity, as compared to other tetracyclines, and ability to combat tetracycline resistance mechanisms such as acquisition of tetracycline-specific efflux pump and protection of ribosomal protein. However, during the last decade, tigecycline resistance has been increasingly reported^9^. To date, several tigecycline-resistance mechanisms among *K. pneumoniae* have been identified. The most common one is chromosomal-mediated overproduction of nonspecific resistance-nodulation-division (RND) efflux pumps such as AcrAB-TolC^10^. In addition, mutations in the *rpsJ* gene, encoding the ribosomal protein S10, have been reported to reduce tigecycline susceptibility^11^. Apart from chromosomal mediated, plasmid-mediated mechanisms such as acquisition of *tet*(A) gene, *tmexCD1-toprJ1* gene cluster, and *tet*(X) gene, which encodes major facilitator superfamily (MFS) efflux pumps, RND-family efflux pump, and tigecycline-modifying enzyme, respectively have also been reported^12–14^. However, the mechanism of tigecycline resistance is not fully understood and additional mechanisms may remain to be discovered.

To unveil the prevalence and mechanisms of tigecycline resistance among C-C-RKP in Thailand, clinical C-C-RKP isolates were obtained from public hospitals. Antimicrobial susceptibility profile, clonal relatedness, and the presence of acquired carbapenemase (*bla*_KPC_, *bla*_NDM_, *bla*_OXA-48-like_*, bla*_IMP_ and *bla*_VIM_), colistin (*mcr*-1 to *mcr*-9), and tigecycline (*tet*(A), *tet*(X) and *tmexCD1-toprJ1*) resistance genes were investigated. For chromosomal-mediated tigecycline resistance, an efflux pump activity was assayed, then expression level of AcrAB efflux pump, and the regulator RamA were measured. Finally, whole genome sequencing was performed to elucidate the mutations associated with tigecycline resistance including AcrR (AcrAB-TolC efflux pump local repressor), RamR, MarR, and SoxR (global regulators repressors), OqxR (OqxAB efflux pump local repressor), Lon protease and *rpsJ* gene.

## Results

### Antimicrobial susceptibility profile

Of 240 C-C-RKP isolates, 220 isolates (91.7%) were resistant to tigecycline, and the MIC_50_ and MIC_90_ of tigecycline were 2 μg/ml and 4 μg/ml, respectively. Among tigecycline-resistant isolates, as shown in Table 1, most isolates exhibited high-level resistance to several last-resort antibiotics. The MIC_50_/MIC_90_ of imipenem, meropenem, colistin, and tigecycline were 256/256, 128/256, 64/128, and 2/4 µg/ml, respectively. Furthermore, most of the isolates were resistant to fosfomycin (70.4%), an important treatment option, with MIC_50_/MIC_90_ of 512/512 µg/ml. However, a large proportion of these isolates remained susceptible to gentamicin (30%), and chloramphenicol (29.1%).

**Table 1:** The resistance rate, MIC_50_, MIC_90_, and MIC range values of 9 antibiotics among tigecycline-resistant C-C-RKP isolates.

Antibiotic	Resistant rate (%)	MIC_50_ (µg/ml)	MIC_90_ (µg/ml)	MIC range (µg/ml)
Amikacin	82.7	32	256	1–512
Chloramphenicol	29.1	16	256	4–256
Colistin	100	64	128	4–1024
Fosfomycin	70.4	512	512	1–1024
Gentamicin	30	2	128	0.25–256
Imipenem	100	256	256	4–512
Meropenem	100	128	256	4–512
Tetracycline	78.2	256	256	2–256
Tigecycline	100	2	4	1–8

### Acquired antibiotic resistant mechanisms

**Figure 1:**
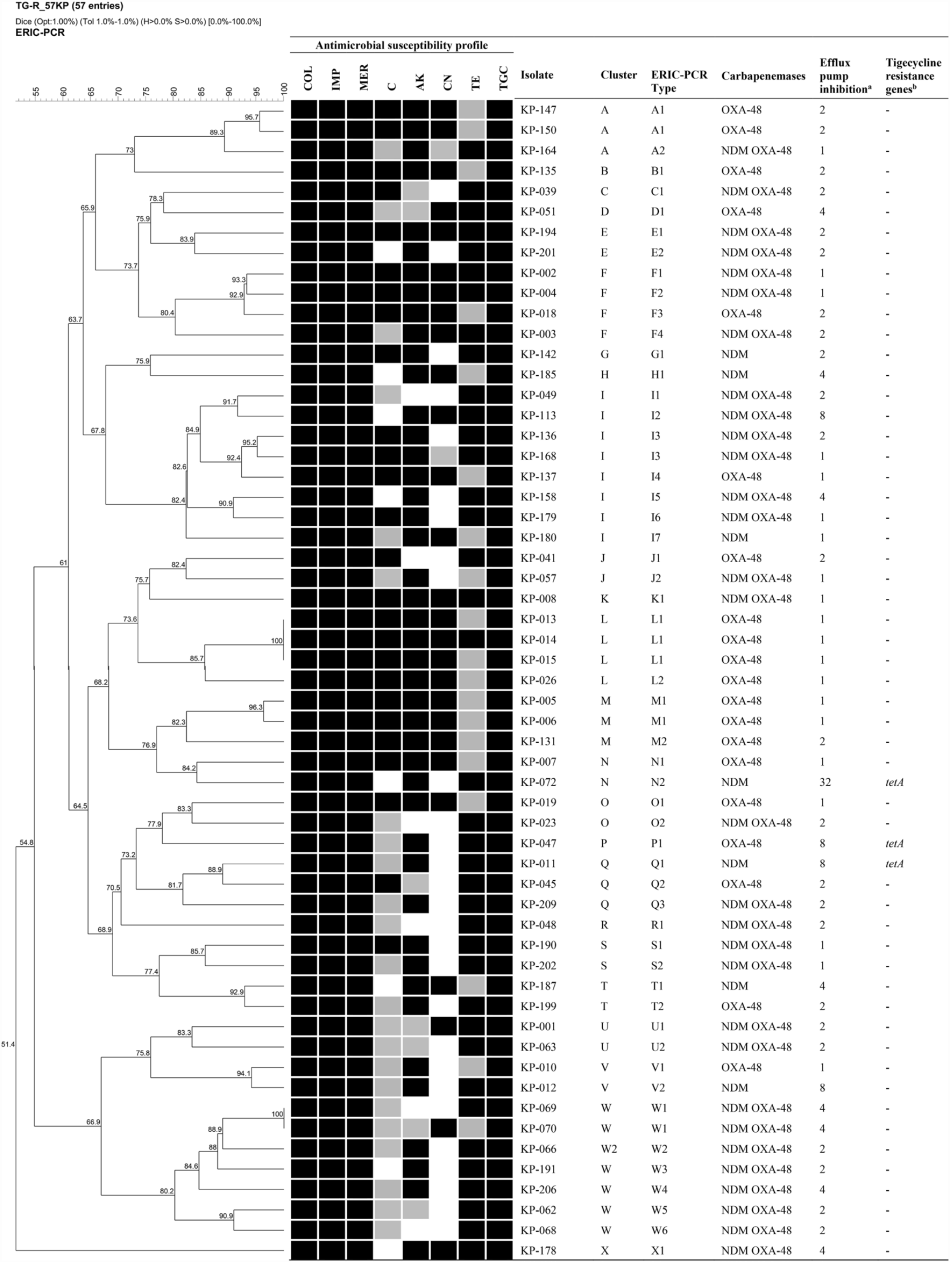
Dendrogram based on ERIC-PCR typing, antimicrobial susceptibility profile, resistance gene profile, and efflux pump activity of 57 C-C-RKP isolates with tigecycline MIC ranged from 4 to 8 µg/ml. The black, gray, and white color in antimicrobial susceptibility profile indicate resistant, intermediate, and susceptible phenotype to an antibiotic, respectively. ^a^Fold reduction of tigecycline MIC in presence of 20 µg/ml CCCP compared to absence of CCCP. ^b^Screening of tigecycline resistant gene including *tet*(A), *tet*(X), and *tmexCD1-toprJ1.*

Based on single-plex and multiplex PCR, tigecycline-resistant isolates were screened for acquired antibiotic resistance determinants including *bla*_KPC,_ *bla*_NDM_, *bla*_IMP_, *bla*_VIM_, *bla*_OXA-48-like_, *mcr*-1–*mcr*-9, *tet*(A), *tet*(X), and *tmexCD1-toprJ1*. The major mechanism of carbapenem resistance was coexistence of *bla*_NDM_ and *bla*_OXA-48-like_ genes (55%) followed by harboring of only *bla*_OXA-48-like_ gene (34.5%) and only *bla*_NDM_ gene (10%), respectively. On the other hand, *bla*_KPC_, *bla*_IMP_, and *bla*_VIM_ genes were not detected. For acquired tigecycline resistance, 4 isolates were found to harbor *tet*(A) gene while *tet*(X) and *tmexCD1-toprJ1* genes were not detected (Fig. 1). None of the isolates were found to carry *mcr* genes.

### Chromosomal-mediated tigecycline resistance

All tigecycline-resistant C-C-RKP isolates (MIC > 0.5 µg/ml) were tested for efflux pump activity. Twenty-nine isolates showed tigecycline MIC reduction ≥ 4-fold in the presence of carbonyl cyanide m-chlorophenylhydrazone (CCCP). This result indicated a positive efflux pump activity (Table S2). Among these isolates, 12 isolates were from high-level tigecycline resistance group (TGC MIC 4–8 µg/ml) while 17 isolates were from low-level tigecycline resistance group (TGC MIC 1–2 µg/ml) (Table S2).

The expression level of AcrAB-TolC efflux pump and the regulator RamA among efflux pump positive isolates were evaluated using qRT-PCR. As compared to *K. pneumoniae* ATCC 13883, the result revealed an alteration in expression level of the efflux pump (*acrB*) and the regulator (*ramA*) (Table S2). Among the high-level tigecycline-resistant group, most isolates showed upregulation of *acrB* (1.2 to 8.1-fold) and upregulation of *ramA* (1.9 to 24.9-fold). Conversely, in the low-level tigecycline-resistant group, expression of *acrB* was slightly increased (1.01 to 2.63-fold) while expression level of *ramA* was diverse (1.50 to 9.83-fold).

**Figure 2:**
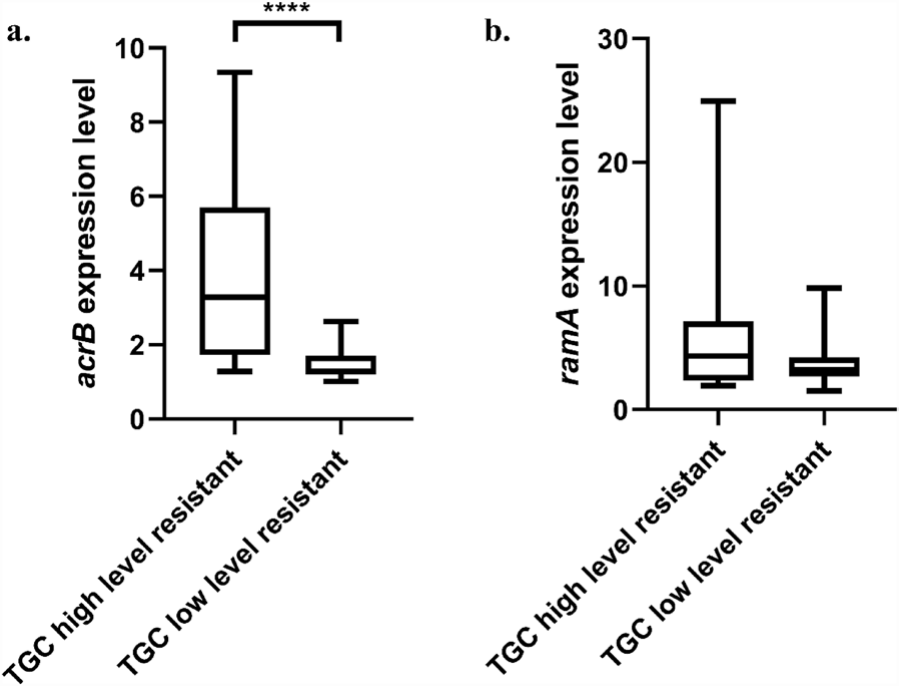
Relative expression level of (**a**) *acrB*, and (**b**) *ramA* of tigecycline high-level resistant isolates (TGC MIC > 2 µg/ml, N = 12), and tigecycline low-level resistant isolates (TGC MIC 1–2 µg/ml, N = 17). ***P = 0.0002; Mann–Whitney* U* test.

The expression levels of *acrB* and *ramA* between the two tigecycline-resistant groups were evaluated using Mann–Whitney *U* test. The result showed that *acrB* expression level was significantly increased in high-level tigecycline-resistant group when compared to that of low-level tigecycline-resistant group (*P* = 0.0002) (Fig. 2a). However, the expression level of *ramA* showed no significant difference between these two groups (*P* = 0.3698) (Fig. 2b).

### Whole genome sequence (WGS) analysis of tigecycline resistant isolates

To identify the causes of tigecycline resistance in C-C-RKP, 15 isolates with positive efflux pump activity were selected and subjected to whole genome sequencing. The analysis focused on mutations in the regions which have been reported to have a correlation with the overexpression of AcrAB-TolC efflux pump, including RamR (*ramA* repressor), PI and PII regions (recognition sites of RamR in *romA-ramA* operon), AcrR (*acrAB* repressor), and Lon protease (*ramA* regulator). Mutations within repressors of the other AcrAB-TolC regulators including MarA and SoxS were also considered. Apart from AcrAB-TolC efflux pump, other mechanisms reported to confer tigecycline resistance including mutations in *rpsJ* gene (a ribosomal S10 encoding gene), mutation in OqxR (OqxAB efflux pump regulator), acquisition of Tet(A) efflux pump, TmexCD1-ToprJ1 efflux pump, and Tet(X) (a tigecycline degrading enzyme) were investigated. WGS analysis revealed that most of the isolates contained mutations within RamR except for XDR-KP-047 and XDR-KP-059 (Table 2). Among high-level tigecycline-resistant isolates, three major patterns of RamR mutations were observed. First, a certain number of isolates were presented with a C-nucleotide insertion in *ramR* gene at position 99 or 100, leading to a frameshift mutation causing premature stop codon at amino acid position 53. These isolates also possessed C139T point mutation in RamR recognition site PI, which might prevent RamR binding leading to the overexpression of *ramA* and *acrB*. Second, certain isolates had S157P mutation in RamR without additional mutations in either RamR recognition site PI or AcrR. Third, a group of isolates possessed co-mutations of RamR and RamR recognition site PI, and/or AcrR. For instance, XDR-KP-113 contained double mutations in RamR (A28T and I141T) and RamR recognition site PI (C139T), XDR-KP-051 carried a single point mutation within both RamR (A20D) and AcrR (W50R), and XDR-KP-206 possessed co-mutation within these three regions.

**Table 2:** The result of multilocus sequence typing (MLST), efflux pump inhibition assay, relative expression level of *acrB* and *ramA*, mutation analysis among regulators of AcrAB-TolC efflux pump, and presence of tigecycline-resistance genes in 15 C-C-RKP isolates.

No.	Project no.	ST	Efflux pump inhibition assay			Gene expression level		Mutation analysis^a^								Tigecycline resistant genes			
			TGC MIC	TGC MIC + CCCP	Fold reduction	*acrB*	*ramA*	RamR	RamR recognition site (PI) (nucleotide sequence)^b^	RamR recognition site (PII) (nucleotide sequence)	AcrR	Lon protease	MarR	SoxR	OqxR	*rpsJ*	*tet*(X)	*tet*(A)	*tmexCD1-toprJ1*
1	XDR-KP-034	16	1	0.25	4	2.63 ± 0.112	3.96 ± 0.384	M184V	–	–	–	–	S82G	–	–	–	–	–	–
2	XDR-KP-100	16	1	0.25	4	1.36 ± 0.113	4.44 ± 0.419	I141T	C139T	–	–	–	S82G	–	–	–	–	–	–
3	XDR-KP-059	147	2	0.125	16	1.50 ± 0.130	1.50 ± 0.385	–	–	–	R13W	–	S82G	–	–	–	–	*tet*(A) (X61367)	–
4	XDR-KP-110	16	2	0.5	4	2.49 ± 0.131	9.83 ± 0.169	Y59C, I141T	C139T	–	–	–	S82G	–	–	–	–	–	–
5	XDR-KP-117	16	2	0.5	4	1.97 ± 0.129	1.94 ± 0.128	I141T	C139T	–	–	–	S82G	–	–	–	–	–	–
6	XDR-KP-189	15	2	0.25	8	1.26 ± 0.170	3.56 ± 0.251	A19V	T131A	–	–	–	S82G	–	–	–	–	–	–
7	XDR-KP-051	101	4	1	4	5.61 ± 0.178	6.31 ± 0.193	A20D	–	–	W50R	–	S82G	–	–	–	–	–	–
8	XDR-KP-069	16	4	1	4	5.79 ± 0.082	24.97 ± 0.136	C99ins- frameshift mutation, E53*	C139T	–	–	–	S82G	–	–	–	–	–	–
9	XDR-KP-070	16	4	1	4	9.34 ± 0.08	3.06 ± 0.223	C100ins- frameshift mutation, E53*	C139T	–	–	–	S82G	–	–	–	–	–	–
10	XDR-KP-072	147	4	0.125	32	1.56 ± 0.173	2.46 ± 0.284	S157P	–	–	–	–	S3N, S82G	–	–	–	–	*tet*(A) (X61367)	–
11	XDR-KP-185	15	4	1	4	1.28 ± 0.326	2.06 ± 0.344	A19V	–	–	–	–	S82G	–	–	–	–	–	–
12	XDR-KP-206	16	4	1	4	4.78 ± 0.039	2.82 ± 0.338	I141T	C139T	–	L154R	–	S82G	–	–	–	–	–	–
13	XDR-KP-011	147	8	1	8	3.29 ± 0.265	5.08 ± 0.337	S157P	–	–	–	–	S3N, S82G	–	–	–	–	*tet*(A) (X61367)	–
14	XDR-KP-047	147	8	1	8	4.82 ± 0.302	10.07 ± 0.043	–	–	–	R13Q	–	S3N, S82G	–	–	–	–	*tet*(A) (X61367)	–
15	XDR-KP-113	16	8	1	8	8.15 ± 0.364	4.33 ± 0.222	A28T, I141T	C139T	–	–	–	S82G	–	–	–	–	–	–

^a^The sequence of the isolates was compared to *K. pneumoniae* ATCC 13883. ^b^Nucleotide numbering system was according to Rosenblum et al.^51^.

Among low-level tigecycline-resistant isolates, most of which also possessed mutations in RamR, and RamR recognition site PI. However, most of these mutations, especially I141T and A19V in RamR and R13W in AcrR, have been reported to have no effects on overexpression of RamA. In addition, mutations within MarR and SoxR, the repressor of MarA and SoxS, respectively, were investigated. Within MarR, as compared to *K. pneumoniae* ATCC 13883, a single point mutation S82G was detected in all isolates and double mutations S3N/S82G were detected in 3 isolates of high-level tigecycline-resistant group. No mutations were detected for SoxR. Apart from an overexpression of AcrAB-TolC efflux pump, four isolates were found to harbor Tet(A) efflux pump. All of them were the mutated Tet(A) protein, which contained 7 mutations including I5R, V55M, I75V, T84A, S201A, F202S, V203F. Two of the four isolates, XDR-KP-011 and XDR-KP-047, also possessed overexpression of *acrB* (Table 2). Thus, co-existence of the mutated Tet(A) efflux pump and overexpression of *acrB* were believed to be the cause of high-level tigecycline resistance (TGC MIC 8 µg/ml) in these two isolates. For the other 2 isolates, XDR-KP-059 and XDR-KP-072, Tet(A) appeared to be solely responsible for tigecycline resistance since the expression of *acrB* was only 1.5-fold upregulated. Mutations in *rpsJ* and OqxR repressor were not found and mobilized tigecycline resistance genes including Tet(X) and TmexCD-ToprJ efflux pump were not detected.

### Clonal relatedness among tigecycline resistant C-C-RKP

The isolates showing tigecycline MIC ranging from 4 to 8 µg/ml were selected to perform ERIC-PCR. According to the result, similarity of genotypic fingerprints among these isolates was ranging from 51.4 to 100%. Clusters were differentiated using 85% similarity, and ERIC types were classified using 95% similarity. Twenty-four clusters were distinguished (cluster A to X) and two major clusters including cluster I (14%) and W (12%) were identified (Fig. 1). Among these clusters, 51 ERIC types were differentiated. The major ERIC types were A1, I3, L1, M1, and W1. The ERIC type L1 contained three indistinguishable C-C-RKP harboring *bla*_OXA-48-like_ gene. All these isolates possessed low MIC of imipenem and meropenem (16 µg/ml). However, these isolates showed high-level resistance to other antibiotics including colistin (MIC 16 µg/ml), chloramphenicol (MIC > 256 µg/ml), amikacin (MIC > 256 µg/ml), gentamicin (MIC > 128 µg/ml), and fosfomycin (MIC > 512 µg/ml). Furthermore, ERIC type L1 members also exhibited high-level tigecycline resistance (MIC 4 µg/ml) but without efflux pump activity. ERIC type I3 and W1 were found to co-harbor *bla*_NDM_ and *bla*_OXA-48-like_ genes. The ERIC type I3 contained two C-C-RKP isolates, XDR-KP-136 and XDR-KP-168, which were collected from different hospitals. Yet, both isolates possessed XDR phenotype with high-level resistance to several antibiotics especially imipenem (MIC > 256 and 64 µg/ml), meropenem (MIC 256 and 64 µg/ml), and colistin (MIC 64 and 32 µg/ml). These isolates also had high-level tigecycline resistance (MIC 4 µg/ml). ERIC type W1 contained two indistinguishable C-C-RKP isolates but different carbapenem-resistance characteristics. XDR-KP-069 and XDR-KP-070 co-harbored *bla*_NDM_ and *bla*_OXA-48-like_ genes. Both isolates displayed high-level of resistance to colistin (MIC 128 µg/ml) caused by mutations in *pmrA* and *pmrB* genes, and to fosfomycin (MIC > 512 µg/ml). These isolates also showed high-level resistance to tigecycline (MIC 4 µg/ml) with an activity of efflux pump. In addition, these isolates possessed a major frameshift mutation in *ramR* and a mutation in RamR recognition site PI, resulting in an overexpression of both *ramA* and *acrB*. These results suggested that the clonal dissemination of chromosomal AcrAB-TolC mediated tigecycline resistance was found among some isolates. However, the dissemination of acquired resistance genes including carbapenemase and *tet*(A) genes using the horizontal gene transfer were detected since same genes were found among the different clusters, especially for *tet*(A), which was identified from different ERIC types.

**Figure 3:**
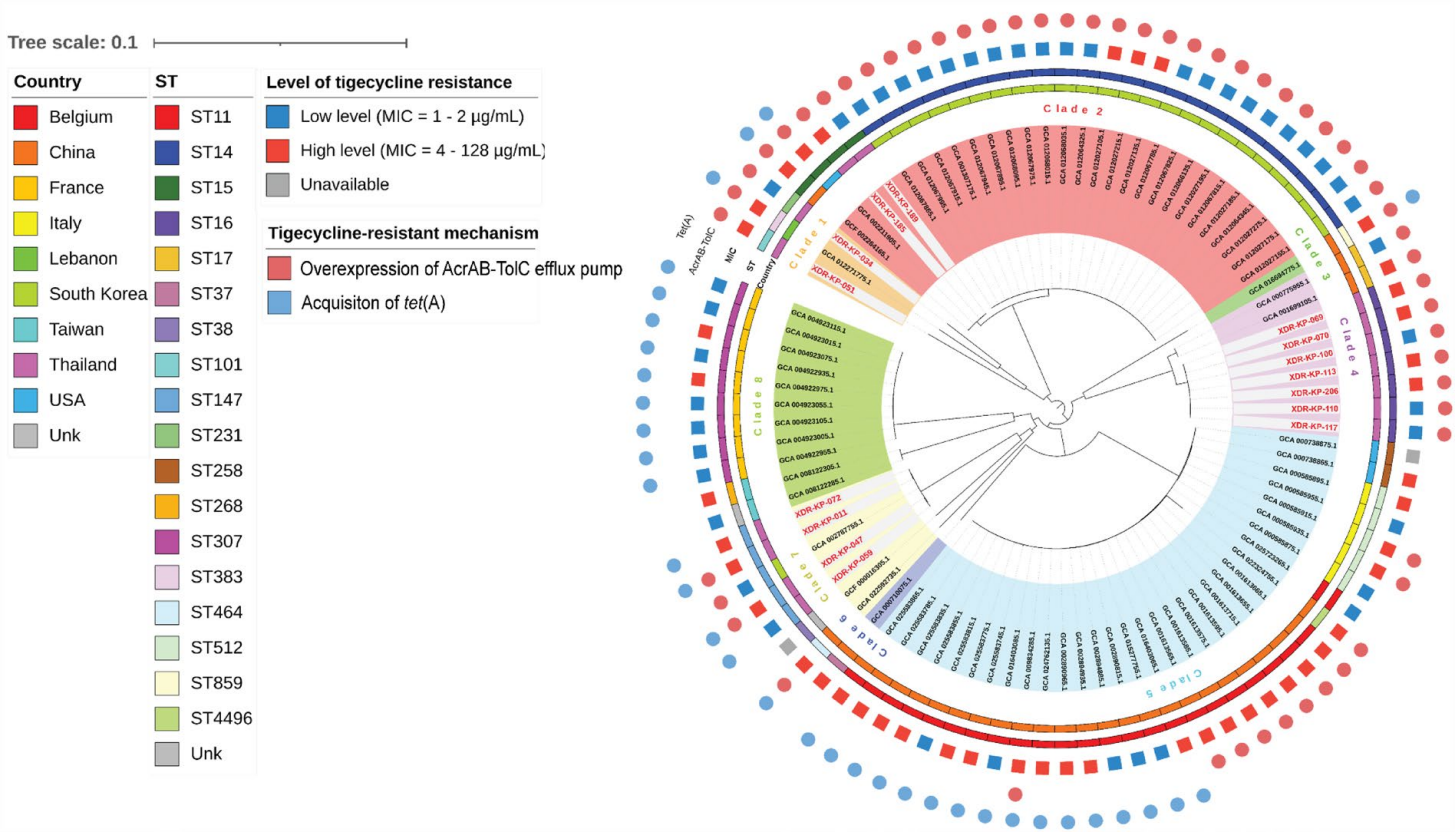
Phylogenetic analysis based on core genome single nucleotide polymorphisms (cgSNP) of 15 C-C-RKP isolates from this study (red labeled) and 76 isolates of tigecycline-resistant *K. pneumoniae* retrieved from NCBI database.

According to the MLST analysis, five STs including ST15, ST16, ST101, ST147, and ST231 were identified. The major STs among tigecycline-resistant C-C-RKP isolates were ST16 and ST147. In ST16, most of the isolates were associated with co-harboring of *bla*_NDM_ and *bla*_OXA-48-like_ genes. For tigecycline resistance, overexpression of *acrB* and *ramA* appeared to be the main mechanism, which was caused by the co-mutations of *ramR* gene, RamR recognition site (PI promoter) and/or *acrR* gene. In ST147, four isolates were identified and most of them harbored only *bla*_NDM_ gene. Interestingly, all isolates were found to harbor the mutated *tet*(A) gene which encoded 7 mutations including I5R, V55M, I75V, T84A, S201A, F202S, V203F, compared to wild-type Tet(A) (GeneBank accession number X00006.1). Based on cgSNP, phylogenetic analysis of all published genomes of tigecycline-resistant *K. pneumoniae* isolates from more than nine countries across the world (15 from this study and 76 from NCBI database) revealed eight major clades (Fig. 3). The MIC levels and mechanisms of tigecycline resistance appeared to be diverse. Notably, the co-existence of AcrAB-TolC overexpression and Tet(A) efflux pump consistently conferred high-level of resistance. The C-C-RKP isolates in our study were classified into 4 clades, including clade 1, 2, 4, and 7. In clade 1, C-C-RKP ST101 and ST231 were closely related to *K. pneumoniae* ST383 clinically isolated from Lebanon^15^. This isolate showed pan drug-resistant (PDR) phenotype, which harbored 47 AMR determinants including carbapenemase genes (*bla*_NDM-5_ and *bla*_OXA-48_), chromosomal-mediated colistin resistance, as well as tigecycline resistance due to mutations in RamR and Tet(A) efflux pump (X61367). All members of clade 2 belonged to clonal group 15, which included OXA-232-producing C-C-RKP ST14 isolates from South Korea^16^, and *K. pneumoniae* ST15 isolates from the United States^17^ and China^18^. Two C-C-RKP ST15 from our study showed close association with clinical isolates from the USA^17^ and China^18^. The isolate from the USA showed PDR phenotype which harbored *bla*_NDM-1_ for carbapenem resistance, disrupted *mgrB* gene for colistin resistance, mutated *ramR* and mutated Tet(A) efflux pump (GeneBank accession number X61367.1) for tigecycline resistance. An additional ST15 strain from China was KPC-2-producing *K. pneumoniae* which was resistant to almost all tested antibiotics except for colistin. This isolate showed high-level tigecycline resistance which was caused by mutations in *ramR* and acquisition of *tet*(A). Clade 4 consisted of our ST16 isolates and *K. pneumoniae* ST17 isolates from China^19^. Lastly, clade 7 comprised all of our ST147, which showed close relationship with a NDM-5 and OXA-181 co-producing *K. pneumoniae* ST147 from South Korea^20^. In contrast to our ST147 isolates, the Korean isolate did not carry a mutated Tet(A) efflux pump. Instead, it exhibited tigecycline resistance due to the insertion of a DNA fragment flanking the RamR recognition site within the *romA* gene. Furthermore, our ST147 isolates shared the same origin with KPC-2 and NDM-1 co-producing *K. pneumoniae* ST464, which also harbored mutated Tet(A) efflux^21^.

## Discussion

The emergence of carbapenem and colistin resistance in *K. pneumoniae* has intensified the antimicrobial resistance problem since few treatment options are left available. Tigecycline is one of the last-resort drugs that has been recommended for treatment of C-C-RKP infection^5^. Nevertheless, tigecycline resistance has been increasingly reported. In this study, a high rate of tigecycline resistance (91.7%) was observed among C-C-RKP, consistent with recent findings in Thailand that tigecycline resistance rate in carbapenem-resistant *K. pneumoniae* ranged from 46.7 to 79.6%^22–24^. However, it is worth noting that a clinical breakpoint of tigecycline resistance for *Klebsiella* spp. is not yet available in CLSI and EUCAST interpretive guidelines. Thus, tigecycline resistance rates in these studies were reported based on EUCAST breakpoints recommended for *E. coli* and *C. koseri* (MIC > 0.5 µg/ml)^25^. In recent years, it has been demonstrated that the dosage regimen of tigecycline could reach the serum concentrations of up to 2 µg/ml^26^ and several surveillance programs have reported tigecycline MIC_90_ at approximately 2 µg/ml among *Klebsiella* isolates^27,28^. Therefore, in accordance with these studies, the isolate with tigecycline MIC greater than 2 µg/ml were considered by US-FDA^29^ and BSAC^30^ as a non-susceptible and a resistant isolate, respectively. Interestingly, although 2 µg/ml was applied, tigecycline resistance rate among C-C-RKP in our study was still high (23.8%). This rate was even higher than that of the other global reports in carbapenem-resistant isolates, ranging from 1.9 to 8.9%^31–35^.

Apart from carbapenems, colistin, and tigecycline, the isolates in our study also showed high rate of resistance to several antibiotics. However, up to 70% of the isolates were still susceptible to gentamicin and chloramphenicol, coherent with the report of the National Antimicrobial Resistance Surveillance Center Thailand (NARST) in 2022^36^ and with the other study^37^ that the susceptibility rates of gentamicin and chloramphenicol among *K. pneumoniae* were higher than 80%. Moreover, several studies has highlighted the potential of these two agents as treatment options against multidrug-resistant *K. pneumoniae* (MDR-KP)^22,38^. Therefore, gentamicin and chloramphenicol seem to be the most effective agents against MDR-KP, in vitro. Treatment of infection using these drugs in combination with β-lactams/β-lactamase inhibitor reserved for MDR bacteria such as ceftazidime-avibactam, meropenem-vaborbactam, and imipenem-relebactam may provide the best treatment outcome^6^.

Characterization of carbapenem-resistance mechanisms in this study revealed that co-carriage of *bla*_NDM_ and *bla*_OXA-48-like_ genes was the most prevalent genotype, followed by *bla*_NDM_ gene, and *bla*_OXA-48-like_ gene, respectively. However, other carbapenemase genes including *bla*_IMP_, *bla*_VIM_, and *bla*_KPC_ were not detected. These findings are in an accord with the results from the previous studies that the prevalence of *K. pneumoniae* carrying *bla*_NDM_ and/or *bla*_OXA-48-like_ genes in Thailand is much higher than that of *bla*_IMP_, *bla*_VIM_ and *bla*_KPC_ genes^39–42^. Noteworthy, *bla*_KPC_ genes in Thailand is extremely rare.

In this study, *acrB* overexpression was found in most tigecycline resistant isolates, most of which were associated with the upregulation of *ramA*. This is in accord with the previous studies. Tigecycline resistance in *K. pneumoniae* has been reported to associate with chromosomal-mediated overexpression of AcrAB-TolC efflux pump^10,43–47^. Expression of the efflux pump genes (*acrA* and *acrB*) has been found to significantly increase in the resistant isolates^43,45,47,48^. In addition, linear correlation between expression level of *acrB* and tigecycline MIC has been reported^43,45^. Generally, the expression of the efflux pump genes is controlled by their local repressor AcrR, and the global activators such as RamA, MarA, and SoxS^10,49^. Therefore, the upregulation of the efflux pump genes has been frequently reported with overexpression of these regulators, especially RamA^12,43,50,51^.

Overexpression of RamA has been reported to be caused by several mechanisms, including dysfunctional mutations in RamR, mutations in RamR binding site within RamA promoter regions, and mutations of Lon protease. *ramR* is a transcriptional repressor gene belonging to the TetR family. It encoded the RamR repressor, which binds to the promoter regions of the *ramA* gene, resulting in transcriptional repression. RamR protein consists of 194 amino acids forming 9 alpha helices protein which separated into N-terminal DNA binding domain (α1 to α3), and C-terminal dimerization domain (α4 to α9)^52^. The loss-of-function mutations in RamR have been reported from several species especially *K. pneumoniae*, which resulted in *ramA* overexpression^12,53,54^. In addition, computational analyses have indicated that mutations in the dimerization domain of RamR can lead to a reduction in its DNA binding affinity in *Salmonella* spp.^55^. In our study, mutations in RamR were detected in both DNA binding and dimerization domains but mostly were point mutations that occurred outside hotspot regions. A frameshift mutation from a single nucleotide insertion was detected in two isolates. Most of the isolates with RamR mutations showed overexpression of RamA. In addition, mutations that have been associated with an inability to induce RamA upregulation, including A19V and I141T were detected^12,43,44,51,53^. Apart from RamR, in this study, the mutation within RamR recognition site was also identified. RamR binds to two regions upfront of *ramA* gene (PI and PII promoters) for regulating activity^51^. Yet only a mutation within the PI promoter region was detected. Notably, a C139T point mutation was identified in most of our isolates, which suggests the presence of a potential hotspot within the PI promoter region. Mutations in Lon protease that has been reported to associate with RamA upregulation were not detected^56^. Nevertheless, there were some isolates that showed no correlation between these two genes; upregulation of *ramA* was found without overexpression of *acrB*. To explain this discrepancy, further investigations are required. Noteworthy, one isolate with high-level tigecycline resistance, XDR-KP-185 (MIC 4 µg/ml), showed only 1.28-fold of *acrB* expression level while known-acquired resistant determinants were not detected. This might indicate an activity of other mechanisms, such as OqxAB or KpgABC efflux pump, which have also been reported to confer tigecycline resistance^50,57,58^. Since tigecycline-resistance mechanisms are complex and may involve several efflux pumps and regulators, enhancing the sensitivity of an efflux pump inhibition assay may require the use of multiple types of efflux pump inhibitors. In addition, it may be beneficial to measure the expression levels of other efflux pumps, such as OqxAB, and the expression levels of other global regulators such as MarA, SoxS, and RarA.

This study identified several important STs among XDR- *K. pneumoniae* isolates, including ST15, ST16, ST101, ST147, and ST231. Also, this was the first characterization of tigecycline-resistance mechanism from these STs in Thailand. In this work, ST16 was identified as the predominant clone. ST16 is globally known for its high-risk profile associated with both hypervirulent and drug-resistant characteristics. To date, the emergence of *K. pneumoniae* ST16 has been reported from several countries, including Denmark^59,60^, France^61^, Italy^62^, United Kingdom^63^, Ireland^63^, Croatia^64^, Bulgaria^65^, Canada^66^, Thailand^67,68^, Vietnam^69^, China^70^, Egypt^71^, and Brazil^72^, which covered 4 of 7 continents. Interestingly, very few studies demonstrated the association between tigecycline resistance and this high-risk clone. Nevertheless, *K. pneumoniae* ST16 has been reported to accumulate various types of antimicrobial resistance determinants^73^ such as *bla*_CTX-M-15_^61^, *bla*_KPC_^74^, *bla*_NDM-5_^60^, *bla*_OXA-48-like_^75^, *bla*_NDM-1_ and *bla*_OXA-232_^62,67,68^, *aac*(*6*′)*-lb-cr*^64^, *aac*(*6*′)*-lb*^67^, *qnrS*^67^, and *mgrB* mutation^67,69^. In our study, the accumulation of many antimicrobial resistance determinants (*bla*_CTX-M-15_, *bla*_NDM-1_, *bla*_OXA-232_, *aadA1*, *aadA2, aac*(*6*′)*-lb-cr*, and *aac*(*6*′)*-lb*) was also detected in C-C-RKP ST16 isolates. To the best of our knowledge, this study was the first to demonstrate the connection between the clonal expansion of *K. pneumoniae* ST16 and a high rate of tigecycline resistance among C-C-RKP isolates. Therefore, our finding could provide potential evidence to support the ongoing evolution of the *K. pneumoniae* ST16 against the last-resort antibiotics, indicating the capability of this high-risk clone to pose a significant public health threat. Another significant ST was ST147, recognized as a successful high-risk clone within *K. pneumoniae* which was reported to be associated with β-lactams and fluoroquinolones resistance^76^. ST147 has been reported globally, with multiple outbreaks associated with this ST, except in Antarctica^4^. It has been found to harbor various antimicrobial resistance genes, such as extended-spectrum β-lactamase, carbapenemase, aminoglycosides resistance, fluoroquinolone resistance, and *tet*(A) gene. In Thailand, ST147 carrying *bla*_NDM_ have been reported in several studies^24,77^. However, the major clonal group of *K. pneumoniae-*CG258, comprising ST11, ST258, and ST512, was not detected in our study^4^.

The only acquired tigecycline-resistance mechanism detected in our study was the Tet(A) efflux pump. Four isolates of ST147 were found to harbor the same mutated Tet(A), which contained 7 mutations (I5R, V55M, I75V, T84A, S201A, F202S, V203F). This type of mutated Tet(A) was similar to that of the previous reports from China and Taiwan^12,21,78^. The mutated Tet(A) has been proved to confer tigecycline resistance via gene cloning experiment, which elevated tigecycline MIC of the susceptible organism up to 8-fold^12,78^. The gene has been reported to be located on plasmids^21,78^, suggesting a possibility of conjugative transfer of this gene to different bacterial species. Moreover, the co-existence of this mutated efflux pump with dysfunctional RamR has been reported to exacerbate tigecycline-resistance level^12^. In our study, the mutated Tet(A) was found to co-exist with *acrB* overexpression, which was found to confer high-level tigecycline resistance (MIC 8 µg/ml) in two isolates. This result suggests that beside dysfunctional RamR, the co-occurrence of the mutated Tet(A) with other resistance mechanisms may also be able to confer high-level tigecycline resistance. To our knowledge, this is the first report of mutated Tet(A) harboring C-C-RKP in Thailand. Therefore, presence of the mutated Tet(A) should be carefully monitored. Interestingly, we found that an efflux pump inhibitor CCCP could re-sensitize the isolates to tigecycline that harbor only Tet(A) resistance mechanism. The presence of 20 µg/ml CCCP could reduce the MIC of tigecycline from 2 and 4 µg/ml to 0.125 µg/ml in XDR-KP-059 and XDR-KP-072, respectively. This re-sensitization of isolates to tigecycline indicates effectiveness of an efflux pump inhibitor which could be a promising choice to combat Tet(A) harboring *K. pneumoniae*.

## Methods

### Bacterial strains

A total of 4179 *K. pneumoniae* clinical isolates were collected from public hospitals including King Chulalongkorn Memorial Hospital, Chulalongkorn University, and the Golden Jubilee Medical Center, Mahidol University, during 2017 to 2021. Of these isolates, 240 C-C-RKP isolates were obtained for further investigation. Bacterial species was identified by biochemical testing and MALDI Biotyper system (Bruker Daltonik, Leipzig, Germany).

### Antimicrobial susceptibility testing

The minimum inhibitory concentration (MIC) of 9 antibiotics including amikacin, chloramphenicol, imipenem, fosfomycin, gentamicin, meropenem, tetracycline, and tigecycline was investigated by agar dilution while that of colistin was measured by broth microdilution, then the results were interpreted according to the Clinical and Laboratory Standards Institute (CLSI, 2023) guidelines^79^. Specifically for tigecycline, resistance cutoff at MIC > 0.5 µg/ml for *E. coli* and *C. koseri* was used according to European Committee on Antimicrobial Susceptibility Testing (EUCAST, 2023) document^25^. *E. coli* ATCC 25922 and *P. aeruginosa* ATCC 27853 were used as reference strains.

### Clonal analysis

Genomic DNA (gDNA) of tigecycline-resistant C-C-RKP was extracted using TIANamp Genomic DNA KIT (TIANGEN, China) according to manufacturer’s instructions. ERIC-PCR was modified according to the protocol published by James and colleagues^80^. The thermal cycling conditions included initial denaturation at 94 °C for 5 min, 35 cycles of denaturation at 94 °C for 1 min, annealing at 38 °C for 1 min, extension at 72 °C for 3 min, and followed by final extension at 72 °C for 10 min. The PCR products were loaded onto a 1.5% agarose gel and ran at 100 V for 1 h, then the result was visualized using gel-docking system. The ERIC pattern was analyzed using InfoQuest FP software version 4.5 by Dice coefficient, unweighted pair group method with arithmetic means (UPGMA) at 1% optimization and 1% band position tolerance. A cluster of the isolates was differentiated at 85% similarity, and an ERIC type was differentiated at 95% similarity. Multilocus sequence typing (MLST) was performed based on 7 housekeeping genes for *K. pneumoniae* including *gapA*, *infB*, *mdh*, *pgi*, *phoE*, *rpoB*, and *tonB*. The allelic number and sequence type (ST) were determined by comparing with MLST database using online PubMLST database^81^.

### Detection of acquired antimicrobial resistance genes

Carbapenemase genes (*bla*_KPC_, *bla*_NDM_, *bla*_IMP_, *bla*_VIM_, and *bla*_OXA-48-like_)^82^ and mobilized colistin resistance genes (*mcr*-1 to *mcr*-9) were detected by multiplex PCR according to our previous studies^83,84^. Acquired tigecycline resistance genes including *tet*(X)^85^, *tet*(A)^86^, and *tmexCD1-toprJ1*^87^ were screened by single-plex PCR. The final 20 µl reaction contained 0.5 µM of each primer, 1.5 mM of MgCl_2_, 200 µM of dNTPs, and 2.5 units of DNA polymerase. The PCR conditions were slightly modified from the original studies. Briefly, the conditions included the initial denaturation at 96 °C for 3 min, 30 cycles of denaturation at 96 °C for 30 s, annealing at 52 °C for *tet*(X), 55 °C for *tmexC1*, and 59 °C for *tet*(A) for 30 s, extension at 72 °C for 30 s, and final extension at 72 °C for 10 min. The primers used for single-plex and multiplex PCR were listed in Table S1.

### Efflux pump inhibition assay

Efflux pump activity was assayed using efflux pump inhibitor, carbonyl cyanide m-chlorophenylhydrazone (CCCP) (TCI, Japan). Briefly, MIC of tigecycline was evaluated using agar dilution method in the presence and absence of 20 µg/ml of CCCP. Then, a 4-fold or greater reduction of tigecycline MIC observed in the presence of CCCP, compared to the absence of CCCP, was considered as positive for efflux pump activity^88^.

### Measurement of AcrAB efflux pump expression level

Quantitative reverse transcription-polymerase chain reaction (qRT-PCR) was used to assess the expression levels of efflux pump gene *acrB*, and transcription activator gene *ramA*. The expression level of *rrsE* housekeeping gene was also measured and served as the reference for normalizing the expression level of each target gene. The specific primers were listed in Table S1^43^. Total bacterial RNA was extracted from log-phase culture using TRNzol Universal Reagent (Tiangen, China), and was treated with DNase I (ThermoFisher Scientific, Waltham, USA). Then, qRT- PCR was performed with KAPA SYBR^®^ FAST One-Step qRT-PCR (KAPA BIOSYSTEMS (PTY) LTD, South Africa). The final 15 µl PCR reaction included 30 ng of DNase treated RNA, 0.4 µM of primers for *acrB* reaction and 0.15 µM of primers for *ramA* and *rrsE* reactions. The PCR condition included cDNA synthesis at 42 °C for 15 min, pre-denaturation at 95 °C for 5 min, and 40 cycles of denaturation at 95 °C for 15 s and annealing at 60 °C for 60 s. The result was analyzed using CFX Maestro software based on the 2^−ΔΔct^ method^43^. Relative expression level of the mRNA was compared to that of *K. pneumoniae* ATCC 13883, a tigecycline-susceptible reference strain.

### Whole genome sequencing

Single colony of the selected C-C-RKP isolates with tigecycline-resistant phenotype was cultured overnight. Then, gDNA was extracted and purified using DNeasy^®^ Blood and Tissue Kit (QIAGEN, Germany). The library preparation was performed using VAHTS Univeral DNA Library Prep kit (Vazyme Biotech, China). The gDNA was sequenced using Illumina Novaseq 6000, PE150 platform (Illumina, San Diego, CA, USA). The quality of raw read was investigated using FastQC tool (Version 0.12.1)^89^. Then, raw data was trimmed using TrimGalore (version 0.6.10)^90^. Bacterial genomes were later assembled using Spades (version 1.1.0) and Quast tool (Version 5.2.0) was performed to assess quality of the assembly^91,92^. Prokka (version 1.14.6) was used to annotate genes^93^. The antimicrobial resistance and plasmid were determined by Staramr tool (version 0.9.1) which cooperated with ResFinder, and PlasmidFinder, respectively^94^. The genomes were compared using the Basic Local Alignment Search Tool (BLAST, NCBI)^95^. The analysis of gene mutation was based on a comparison of the C-C-RKP′ genes to the reference sequence *K. pneumoniae* subsp. pneumoniae MGH 78578 (GeneBank accession number CP000647), and to wild-type *K. pneumoniae* ATCC 13883 (GeneBank accession number NZ_JSZI00000000).

### Phylogenetic analysis

Core genome single nucleotide polymorphisms (cgSNP) was performed to determine the number of core genome SNP from draft genomes of tigecycline-resistant C-C-RKP clinical isolates in Thailand and from varying tigecycline-resistant *K. pneumoniae* WGS investigations conducted elsewhere (available on NCBI database). *K. pneumoniae* subsp. pneumoniae MGH 78578 (NCBI RefSeq assembly GCF_000016305.1) was used as a reference to generate a core genome alignment and phylogenetic tree was constructed using a core SNP alignment. Draft genomes of *K. pneumoniae* were aligned following the detection and filtration of recombinant regions using Parsnp v1.2^96^ and Gubbins v2.4.1^97^. Maximum-likelihood (ML) trees were generated by RAxML v8.2.12^98^ using ASC_GTRGAMMA model of rate heterogeneity with the Lewis correction for ascertainment bias^99,100^. Branch support was performed by 1000 bootstrap replicates. Best-scoring ML tree was visualized and annotated as a phylogenetic tree using FigTree v1.4.4 and Evolview v2^101,102^.

### Statistical analysis

Statistical analysis of an association between tigecycline-resistance level and expression level of *acrB* and *ramA* was performed using GraphPad Prism 8 software. Since the data were not normally distributed, an intergroup comparison was performed using the Mann–Whitney *U* test. A *P* value < 0.05 was considered as statistical significance. The correlation between expression level of *acrB* and tigecycline MIC was evaluated using linear regression.

### Supplementary Information

Supplementary Tables.

## Data Availability

The genomic data of 15 C-C-RKP isolates are available in the NCBI database under BioProject No. PRJNA1000742.

## References

[1] Ten health issues WHO will tackle this year. https://www.who.int/news-room/spotlight/ten-threats-to-global-health-in-2019

[2] Murray CJL (2022). Global burden of bacterial antimicrobial resistance in 2019: A systematic analysis. Lancet.

[3] O’Neill, J. *Tackling Drug-Resistant Infections Globally: Final Report and Recommendations*. https://apo.org.au/node/63983 (2016).

[4] Navon-Venezia S, Kondratyeva K, Carattoli A (2017). *Klebsiella pneumoniae*: A major worldwide source and shuttle for antibiotic resistance. FEMS Microbiol. Rev..

[5] Braykov NP, Eber MR, Klein EY, Morgan DJ, Laxminarayan R (2013). Trends in resistance to carbapenems and third-generation cephalosporins among clinical isolates of *Klebsiella pneumoniae* in the United States, 1999–2010. Infect. Control Hosp. Epidemiol..

[6] Petrosillo N, Taglietti F, Granata G (2019). Treatment options for colistin resistant *Klebsiella pneumoniae*: Present and future. J. Clin. Med..

[7] Sum P-E, Petersen P (1999). Synthesis and structure-activity relationship of novel glycylcycline derivatives leading to the discovery of GAR-936. Bioorg. Med. Chem. Lett..

[8] Yaghoubi S (2022). Tigecycline antibacterial activity, clinical effectiveness, and mechanisms and epidemiology of resistance: Narrative review. Eur. J. Clin. Microbiol. Infect. Dis..

[9] Pournaras S, Koumaki V, Spanakis N, Gennimata V, Tsakris A (2016). Current perspectives on tigecycline resistance in Enterobacteriaceae: Susceptibility testing issues and mechanisms of resistance. Int. J. Antimicrob. Agents.

[10] Ruzin A, Visalli MA, Keeney D, Bradford PA (2005). Influence of transcriptional activator RamA on expression of multidrug efflux pump AcrAB and tigecycline susceptibility in *Klebsiella pneumoniae*. Antimicrob. Agents Chemother..

[11] He F (2018). Tigecycline resistance caused by *rpsJ* evolution in a 59-year-old male patient infected with KPC-producing *Klebsiella pneumoniae* during tigecycline treatment. Infect. Genet. Evol. J. Mol. Epidemiol. Evol. Genet. Infect. Dis..

[12] Chiu S-K (2017). Roles of *ramR* and *tet*(A) mutations in conferring tigecycline resistance in carbapenem-resistant *Klebsiella pneumoniae* clinical isolates. Antimicrob. Agents Chemother..

[13] Lv L (2020). Emergence of a plasmid-encoded resistance-nodulation-division efflux pump conferring resistance to multiple drugs, including tigecycline, *Klebsiella*
*pneumoniae*. mBio.

[14] Zhai W (2022). Presence of mobile tigecycline resistance gene *tet*(X4) in clinical *Klebsiella pneumoniae*. Microbiol. Spectr..

[15] Sleiman A (2021). An unequivocal superbug: PDR *Klebsiella pneumoniae* with an arsenal of resistance and virulence factor genes. J. Infect. Dev. Ctries..

[16] Kim Y-J, Kim S, Kim J, Bae S (2020). Tracking short-term changes in the genetic diversity and antimicrobial resistance of OXA-232-producing *Klebsiella pneumoniae* ST14 in clinical settings. Clin. Microbiol. Infect. Off. Publ. Eur. Soc. Clin. Microbiol. Infect. Dis..

[17] de Man TJB (2018). Genomic analysis of a pan-resistant isolate of *Klebsiella pneumoniae*, United States 2016. mBio.

[18] Xu J, Gao M, Hong Y, He F (2017). Draft genome sequence of a tigecycline-resistant KPC-2-producing *Klebsiella pneumoniae* ST15 clinical isolate from China. J. Glob. Antimicrob. Resist..

[19] Fang L (2016). Step-wise increase in tigecycline resistance in *Klebsiella pneumoniae* associated with mutations in *ramR*, *lon* and *rpsJ*. PLoS One.

[20] Yoon E-J, Oh Y, Jeong SH (2020). Development of tigecycline resistance in carbapenemase-producing *Klebsiella pneumoniae* sequence type 147 via AcrAB overproduction mediated by replacement of the *ramA* promoter. Ann. Lab. Med..

[21] Hao J (2022). Emergence of a hypervirulent tigecycline-resistant *Klebsiella pneumoniae* strain co-producing *bla*_NDM__-1_ and *bla*_KPC__-2_ with an uncommon sequence type ST464 in Southwestern China. Front. Microbiol..

[22] Nulsopapon P (2021). The synergistic activity and optimizing doses of tigecycline in combination with aminoglycosides against clinical carbapenem-resistant *Klebsiella pneumoniae* isolates. Antibiotics (Basel, Switzerland).

[23] Nulsopapon P (2022). Antimicrobial activity profiles and potential antimicrobial regimens against carbapenem-resistant Enterobacterales isolated from multi-centers in Western Thailand. Antibiotics (Basel, Switzerland).

[24] Assawatheptawee K (2023). Presence and characterization of *bla*_NDM__-1_-positive carbapenemase-producing *Klebsiella pneumoniae* from outpatients in Thailand. J. Microbiol. Immunol Infect. Wei Mian Yu Gan Ran Za Zhi.

[25] The European Committee on Antimicrobial Susceptibility Testing. Breakpoint tables for interpretation of MICs and zone diameters, version 13.1 (2023).

[26] Rodvold KA (2006). Serum, tissue and body fluid concentrations of tigecycline after a single 100 mg dose. J. Antimicrob. Chemother..

[27] Dowzicky MJ, Park CH (2008). Update on antimicrobial susceptibility rates among gram-negative and gram-positive organisms in the United States: Results from the Tigecycline Evaluation and Surveillance Trial (TEST) 2005 to 2007. Clin. Ther..

[28] Bouchillon SK, Iredell JR, Barkham T, Lee K, Dowzicky MJ (2009). Comparative in vitro activity of tigecycline and other antimicrobials against Gram-negative and Gram-positive organisms collected from the Asia-Pacific Rim as part of the Tigecycline Evaluation and Surveillance Trial (TEST). Int. J. Antimicrob. Agents.

[29] U.S. Food and Drug administration. Antibacterial Susceptibility Test Interpretive Criteria. https://www.fda.gov/drugs/development-resources/antibacterial-susceptibility-test-interpretive-criteria

[30] British Society for Antimicrobial Chemotherapy. Standing Committee on Susceptibility Testing. version 14.0 (2015).

[31] Sader HS, Flamm RK, Jones RN (2013). Tigecycline activity tested against antimicrobial resistant surveillance subsets of clinical bacteria collected worldwide (2011). Diagn. Microbiol. Infect. Dis..

[32] Hoban DJ, Reinert RR, Bouchillon SK, Dowzicky MJ (2015). Global in vitro activity of tigecycline and comparator agents: Tigecycline Evaluation and Surveillance Trial 2004–2013. Ann. Clin. Microbiol. Antimicrob..

[33] Sader HS, Farrell DJ, Jones RN (2011). Tigecycline activity tested against multidrug-resistant Enterobacteriaceae and *Acinetobacter* spp. isolated in US medical centers (2005–2009). Diagn. Microbiol. Infect. Dis..

[34] Hsu M-S (2011). In vitro susceptibilities of clinical isolates of ertapenem-non-susceptible Enterobacteriaceae to nemonoxacin, tigecycline, fosfomycin and other antimicrobial agents. Int. J. Antimicrob. Agents.

[35] Sader HS (2015). Tigecycline activity tested against carbapenem-resistant Enterobacteriaceae from 18 European nations: Results from the SENTRY surveillance program (2010–2013). Diagn. Microbiol. Infect. Dis..

[36] NARST: National Antimicrobial Resistance Surveillance Center, THAILAND. Antibiograms (2022).

[37] Santimaleeworagun W (2020). The prevalence of colistin-resistant Gram-negative bacteria isolated from hospitalized patients with bacteremia. J. Appl. Pharm. Sci..

[38] Sood S (2016). Chloramphenicol—A potent armament against multi-drug resistant (MDR) gram negative bacilli?. J. Clin. Diagn. Res. JCDR.

[39] Paveenkittiporn W (2021). Molecular epidemiology of carbapenem-resistant Enterobacterales in Thailand, 2016–2018. Antimicrob. Resist. Infect. Control.

[40] Yungyuen T (2021). Nationwide surveillance and molecular characterization of critically drug-resistant gram-negative bacteria: Results of the Research University Network Thailand Study. Antimicrob. Agents Chemother..

[41] Takeuchi D (2022). Nationwide surveillance in Thailand revealed genotype-dependent dissemination of carbapenem-resistant Enterobacterales. Microb. Genom..

[42] Berrazeg M (2014). New Delhi Metallo-beta-lactamase around the world: An eReview using Google Maps. Euro Surveill. Bull. Eur. Sur Mal. Transm. Eur. Commun. Dis. Bull..

[43] Wang X (2015). Genetic characterisation of clinical *Klebsiella pneumoniae* isolates with reduced susceptibility to tigecycline: Role of the global regulator RamA and its local repressor RamR. Int. J. Antimicrob. Agents.

[44] Sheng Z-K (2014). Mechanisms of tigecycline resistance among *Klebsiella pneumoniae* clinical isolates. Antimicrob. Agents Chemother..

[45] He F (2015). Tigecycline susceptibility and the role of efflux pumps in tigecycline resistance in KPC-producing *Klebsiella pneumoniae*. PLoS One.

[46] Chiu S-K (2017). Tigecycline resistance among carbapenem-resistant *Klebsiella pneumoniae*: Clinical characteristics and expression levels of efflux pump genes. PLoS One.

[47] Park Y, Choi Q, Kwon GC, Koo SH (2020). Molecular epidemiology and mechanisms of tigecycline resistance in carbapenem-resistant *Klebsiella pneumoniae* isolates. J. Clin. Lab. Anal..

[48] Elgendy SG, Abdel Hameed MR, El-Mokhtar MA (2018). Tigecycline resistance among *Klebsiella pneumoniae* isolated from febrile neutropenic patients. J. Med. Microbiol..

[49] Veleba M, Schneiders T (2012). Tigecycline resistance can occur independently of the *ramA* gene in *Klebsiella pneumoniae*. Antimicrob. Agents Chemother..

[50] Xu Q (2021). RamA upregulates multidrug resistance efflux pumps AcrAB and OqxAB in *Klebsiella pneumoniae*. Int. J. Antimicrob. Agents.

[51] Rosenblum R, Khan E, Gonzalez G, Hasan R, Schneiders T (2011). Genetic regulation of the *ramA* locus and its expression in clinical isolates of *Klebsiella pneumoniae*. Int. J. Antimicrob. Agents.

[52] Yamasaki S (2013). The crystal structure of multidrug-resistance regulator RamR with multiple drugs. Nat. Commun..

[53] Hentschke M, Wolters M, Sobottka I, Rohde H, Aepfelbacher M (2010). *ramR* mutations in clinical isolates of *Klebsiella pneumoniae* with reduced susceptibility to tigecycline. Antimicrob. Agents Chemother..

[54] Mao Y, Shi Q, Zhang P, Jiang Y, Yu Y (2020). Effect of *ramR* loss-of-function insertion on tigecycline resistance in clinical isolates of carbapenem-resistant *Klebsiella pneumoniae*. J. Glob. Antimicrob. Resist..

[55] Liu Y-Y, Chen C-C (2017). Computational analysis of the molecular mechanism of RamR mutations contributing to antimicrobial resistance in *Salmonella*
*enterica*. Sci. Rep..

[56] Ricci V, Blair JMA, Piddock LJV (2014). RamA, which controls expression of the MDR efflux pump AcrAB-TolC, is regulated by the Lon protease. J. Antimicrob. Chemother..

[57] Zhong X (2014). First emergence of *acrAB* and *oqxAB* mediated tigecycline resistance in clinical isolates of *Klebsiella pneumoniae* pre-dating the use of tigecycline in a Chinese hospital. PLoS One.

[58] Veleba M, Higgins PG, Gonzalez G, Seifert H, Schneiders T (2012). Characterization of RarA, a novel AraC family multidrug resistance regulator in *Klebsiella pneumoniae*. Antimicrob. Agents Chemother..

[59] Nielsen JB (2011). Identification of CTX-M15-, SHV-28-producing *Klebsiella pneumoniae* ST15 as an epidemic clone in the Copenhagen area using a semi-automated Rep-PCR typing assay. Eur. J. Clin. Microbiol. Infect. Dis. Off. Publ. Eur. Soc. Clin. Microbiol..

[60] Bathoorn E, Rossen JW, Lokate M, Friedrich AW, Hammerum AM (2015). Isolation of an NDM-5-producing ST16 *Klebsiella pneumoniae* from a Dutch patient without travel history abroad, August 2015. Euro Surveill. Bull. Eur. Sur Mal. Transm. Eur. Commun. Dis. Bull..

[61] Marcade G (2013). The emergence of multidrug-resistant *Klebsiella pneumoniae* of international clones ST13, ST16, ST35, ST48 and ST101 in a teaching hospital in the Paris region. Epidemiol. Infect..

[62] Avolio M, Vignaroli C, Crapis M, Camporese A (2017). Co-production of NDM-1 and OXA-232 by ST16 *Klebsiella pneumoniae*, Italy, 2016. Future Microbiol..

[63] Moradigaravand D, Martin V, Peacock SJ, Parkhill J (2017). Evolution and epidemiology of multidrug-resistant *Klebsiella pneumoniae* in the United Kingdom and Ireland. mBio.

[64] Kocsis E (2016). *bla*_NDM__-1_ carriage on IncR plasmid in Enterobacteriaceae strains. Microb. Drug Resist. Larchmt. N.

[65] Savov E (2018). NDM-1 hazard in the Balkan states: Evidence of the first outbreak of NDM-1-producing *Klebsiella pneumoniae* in Bulgaria. Microb. Drug Resist. Larchmt. N.

[66] Mulvey MR, Grant JM, Plewes K, Roscoe D, Boyd DA (2011). New Delhi metallo-β-lactamase in *Klebsiella pneumoniae* and *Escherichia coli*, Canada. Emerg. Infect. Dis..

[67] Boonyasiri A (2021). Genomic and clinical characterisation of multidrug-resistant carbapenemase-producing ST231 and ST16 *Klebsiella pneumoniae* isolates colonising patients at Siriraj hospital, Bangkok, Thailand from 2015 to 2017. BMC Infect. Dis..

[68] Abe R (2022). Clonal dissemination of carbapenem-resistant *Klebsiella pneumoniae* ST16 co-producing NDM-1 and OXA-232 in Thailand. JAC-Antimicrob. Resist..

[69] Nguyen TNT (2021). Emerging carbapenem-resistant *Klebsiella pneumoniae* sequence type 16 causing multiple outbreaks in a tertiary hospital in southern Vietnam. Microb. Genom..

[70] Zhang B (2022). Comparison of two distinct subpopulations of *Klebsiella pneumoniae* ST16 co-occurring in a single patient. Microbiol. Spectr..

[71] Sherif M (2021). Whole-genome sequencing of Egyptian multidrug-resistant *Klebsiella pneumoniae* isolates: A multi-center pilot study. Eur. J. Clin. Microbiol. Infect. Dis. Off. Publ. Eur. Soc. Clin. Microbiol..

[72] Seki LM (2011). Molecular epidemiology of KPC-2-producing *Klebsiella pneumoniae* isolates in Brazil: The predominance of sequence type 437. Diagn. Microbiol. Infect. Dis..

[73] de Sales RO, Leaden L, Migliorini LB, Severino P (2022). A Comprehensive genomic analysis of the emergent *Klebsiella pneumoniae* ST16 lineage: Virulence, antimicrobial resistance and a comparison with the clinically relevant ST11 strain. Pathog. Basel Switz..

[74] Andrey DO (2020). An emerging clone, *Klebsiella pneumoniae* carbapenemase 2-producing *K. pneumoniae* sequence type 16, associated with high mortality rates in a CC258-endemic setting. Clin. Infect..

[75] Espinal P (2019). Genomics of *Klebsiella pneumoniae* ST16 producing NDM-1, CTX-M-15, and OXA-232. Clin. Microbiol. Infect. Off. Publ. Eur. Soc. Clin. Microbiol. Infect. Dis..

[76] Peirano G, Chen L, Kreiswirth BN, Pitout JDD (2020). Emerging antimicrobial-resistant high-risk *Klebsiella pneumoniae* clones ST307 and ST147. Antimicrob. Agents Chemother..

[77] Kk S (2021). Optical DNA mapping of plasmids reveals clonal spread of carbapenem-resistant *Klebsiella pneumoniae* in a Large Thai Hospital. Antibiotics.

[78] Yao H (2020). Molecular characterization of an IncFIIk plasmid co-harboring *bla*_IMP__-26_ and *tet*(A) variant in a clinical *Klebsiella pneumoniae* isolate. Front. Microbiol..

[79] CLSI. Performance Standards for Antimicrobial Susceptibility Testing. 33rd edn. CLSI supplement M100 (2023).

[80] Versalovic J, Koeuth T, Lupski R (1991). Distribution of repetitive DNA sequences in eubacteria and application to finerpriting of bacterial enomes. Nucleic Acids Res..

[81] Jolley KA, Bray JE, Maiden MCJ (2018). Open-access bacterial population genomics: BIGSdb software, the PubMLST.org website and their applications. Wellcome Open Res..

[82] Poirel L, Walsh TR, Cuvillier V, Nordmann P (2011). Multiplex PCR for detection of acquired carbapenemase genes. Diagn. Microbiol. Infect. Dis..

[83] Phuadraksa T, Wichit S, Arikit S, Songtawee N, Yainoy S (2022). Co-occurrence of *mcr*-2 and *mcr*-3 genes on chromosome of multidrug-resistant *Escherichia coli* isolated from healthy individuals in Thailand. Int. J. Antimicrob. Agents.

[84] Phuadraksa T (2022). Emergence of plasmid-mediated colistin resistance *mcr*-3.5 gene in *Citrobacter*
*amalonaticus* and *Citrobacter*
*sedlakii* isolated from healthy individual in Thailand. Front. Cell. Infect. Microbiol..

[85] Hsieh Y-C (2021). An outbreak of *tet*(X6)-carrying tigecycline-resistant *Acinetobacter baumannii* isolates with a new capsular type at a hospital in Taiwan. Antibiotics (Basel, Switzerland).

[86] Bokaeian, M., Saeidi, S., Shahi, Z. & Kadaei, V. *tet*(A) and *tet*(B) genes in *Klebsiella pneumoniae* isolated from clinical samples. *Gene Cell Tissue* **1** (2014).

[87] Wan M, Gao X, Lv L, Cai Z, Liu J-H (2021). IS26 mediates the acquisition of tigecycline resistance gene cluster *tmexCD1-toprJ1* by IncHI1B-FIB plasmids in *Klebsiella pneumoniae* and *Klebsiella*
*quasipneumoniae* from food market sewage. Antimicrob. Agents Chemother..

[88] Sanchez-Carbonel A (2021). The effect of the efflux pump inhibitor carbonyl cyanide m-chlorophenylhydrazone (CCCP) on the susceptibility to imipenem and cefepime in clinical strains of *Acinetobacter*
*baumannii*. PLoS One.

[89] Babraham Bioinformatics—FastQC A Quality Control tool for High Throughput Sequence Data. https://www.bioinformatics.babraham.ac.uk/projects/fastqc/

[90] Martin M (2011). Cutadapt removes adapter sequences from high-throughput sequencing reads. EMBnet.journal.

[91] Bankevich A (2012). SPAdes: A new genome assembly algorithm and its applications to single-cell sequencing. J. Comput. Biol. J. Comput. Mol. Cell Biol..

[92] Mikheenko A, Prjibelski A, Saveliev V, Antipov D, Gurevich A (2018). Versatile genome assembly evaluation with QUAST-LG. Bioinformatics (Oxf., Engl.).

[93] Seemann T (2014). Prokka: Rapid prokaryotic genome annotation. Bioinforma. Oxf. Engl..

[94] GitHub—phac-nml/staramr: Scans genome contigs against the ResFinder, PlasmidFinder, and PointFinder databases. https://github.com/phac-nml/staramr

[95] Altschul SF, Gish W, Miller W, Myers EW, Lipman DJ (1990). Basic local alignment search tool. J. Mol. Biol..

[96] Treangen TJ, Ondov BD, Koren S, Phillippy AM (2014). The Harvest suite for rapid core-genome alignment and visualization of thousands of intraspecific microbial genomes. Genome Biol..

[97] Croucher NJ (2015). Rapid phylogenetic analysis of large samples of recombinant bacterial whole genome sequences using Gubbins. Nucleic Acids Res..

[98] Stamatakis A (2014). RAxML version 8: A tool for phylogenetic analysis and post-analysis of large phylogenies. Bioinformatics (Oxf., Engl.).

[99] Yang Z (1996). Among-site rate variation and its impact on phylogenetic analyses. Trends Ecol. Evol..

[100] Leaché AD, Banbury BL, Felsenstein J, de Oca AN-M, Stamatakis A (2015). Short tree, long tree, right tree, wrong tree: New acquisition bias corrections for inferring SNP phylogenies. Syst. Biol..

[101] Zhang H, Gao S, Lercher MJ, Hu S, Chen W-H (2012). EvolView, an online tool for visualizing, annotating and managing phylogenetic trees. Nucleic Acids Res..

[102] He Z (2016). Evolview v2: An online visualization and management tool for customized and annotated phylogenetic trees. Nucleic Acids Res..

