# Supplementary material for: Occurrence and mechanisms of tigecycline resistance in carbapenem- and colistin-resistant Klebsiella pneumoniae in Thailand
Source: Sci Rep. 2024 Mar 3;14:5215. doi: 10.1038/s41598-024-55705-2 (PMC10909888; doi:10.1038/s41598-024-55705-2)
Supplement: Supplementary file 1 — Supplementary Tables. [file 41598_2024_55705_MOESM1_ESM.docx]

**Supplementary information**

**Table S1.** Primers used in this study

| **No.** | **Target gene** | **Primer name** | **Sequence 5' - 3'** | **Annealing temperature (°C)** | | **Target size (bp)** | | **Utilization** | | **Ref.** |
| --- | --- | --- | --- | --- | --- | --- | --- | --- | --- | --- |
| 1 | ERIC | ERIC1 | ATG TAA GCT CCT GGG GAT TCAC | 38 | |  | | DNA fingerprinting | | [65] |
|  |  | ERIC2 | AAG TAA GTG ACT GGG GTG AGC G |  |  |  |  |  |  |  |
| 2 | *tet*(X) | *tet*(X) forward | TTC AGG TCA GGA AGC AAT GAA AA | 52 | | 780 | | PCR | | [70] |
|  |  | *tet*(X) reverse | TTT ACG CCT TGT CCT GCA AAA GG |  |  |  |  |  |  |  |
| 3 | *acrB* | *acrB* forward | AAA CTT CGC CAC TAC GTC ATA | 60 | | 158 | | qRT-PCR | | [39] |
|  |  | *acrB* reverse | AGC TTA ACG CCT CGA TCA T |  |  |  |  |  |  |  |
| 4 | *ramA* | *ramA* forward | GAT ATC GCT CGC CAT GC | 60 | | 251 | | qRT-PCR | |  |
|  |  | *ramA* reverse | CTG TGG TTC TCT TTG CGG TAG |  |  |  |  |  |  |  |
| 5 | *rrsE* | *rrsE* forward | GTC ATC ATG GCC CTT ACG AG | 60 | | 94 | | qRT-PCR | |  |
|  |  | *rrsE* reverse | ACT TTA TGA GGT CCG CTT GCT |  |  |  |  |  |  |  |
| 6 | *tet*(A) | *tet*(A) forward | GTG AAA CCC AAC ATA CCC C | 59 | | 888 | | PCR | | [71] |
|  |  | *tet*(A) reverse | GAA GGC AAG CAG GAT GTA G |  |  |  |  |  |  |  |
| 7 | *tmexC*1 | *tmexC*1 forward | TTC CGT GAT CTC CTG TTT G | 55 | | 880 | | PCR | | [72] |
|  |  | *tmexC*1 reverse | GAT GGC GTT CTG GTT GAG |  |  |  |  |  |  |  |
| 8 | *bla*_KPC_ | KPC forward | CGT CTA GTT CTG CTG TCT TG | 52 | | 789 | | Multiplex PCR | | [67] |
|  |  | KPC reverse | CTT GTC ATC CTT GTT AGG CG |  |  |  |  |  |  |  |
| 9 | *bla*_NDM_ | NDM forward | GGT TTG GCG ATC TGG TTT TC |  |  | 621 | |  |  |  |
|  |  | NDM reverse | CGG AAT GGC TCA TCA CGA TC |  |  |  |  |  |  |  |
| 10 | *bla*_OXA-48-like_ | OXA forward | GCG TGG TTA AGG ATG AAC AC |  |  | 438 | |  |  |  |
|  |  | OXA reverse | CAT CAA GTT CAA CCC AAC CG |  |  |  |  |  |  |  |
| 11 | *bla*_IMP_ | IMP forward | GGA ATA GAG TGG CTT AAY TCT C | 52 | | 232 | |  |  |  |
|  |  | IMP reverse | GGT TTA AYA AAA CAA CCA CC |  |  |  |  |  |  |  |
| 12 | *bla*_VIM_ | VIM forward | GAT GGT GTT TGG TCG CAT A |  |  | 390 | |  |  |  |
|  |  | VIM reverse | CGA ATG CGC AGC ACC AG |  |  |  |  |  |  |  |
| 13 | *mcr-*1 | *mcr-*1 forward | ATG CCA GTT TCT TTC GCG TG | 59 | | 502 | | Multiplex PCR | | [68,69] |
|  |  | *mcr*-1 reverse | TCG GCA AAT TGC GCT TTT GGC |  |  |  |  |  |  |  |
| 14 | *mcr-*2 | *mcr-*2 forward | GAT GGC GGT CTA TCC TGT AT |  |  | 379 | |  |  |  |
|  |  | *mcr*-2 reverse | AAG GCT GAC ACC CCA TGT CAT |  |  |  |  |  |  |  |
| 15 | *mcr-*3 | *mcr-*3 forward | ACC AGT AAA TCT GGT GGC GT |  |  | 296 | |  |  |  |
|  |  | *mcr*-3 reverse | AGG ACA ACC TCG TCA TAG CA |  |  |  |  |  |  |  |
| 16 | *mcr-*4 | *mcr-*4 forward | TTG CAG ACG CCC ATG GAA TA |  |  | 207 | |  |  |  |
|  |  | *mcr*-4 reverse | GCC GCA TGA GCT AGT ATC GT |  |  |  |  |  |  |  |
| 17 | *mcr-*5 | *mcr-*5 forward | GGA CGC GAC TCC CTA ACT TC |  |  | 608 | |  |  |  |
|  |  | *mcr*-5 reverse | ACA ACC AGT ACG AGA GCA CG |  |  |  |  |  |  |  |
| 19 | *mcr-*6 | *mcr-*6 forward | AGC TAT GTC AAT CCC GTG AT | | 55 | | 252 | | Multiplex PCR | [68,69] |
|  |  | *mcr*-6 reverse | ATT GGC TAG GTT GTC AAT C | |  |  |  | |  |  |
| 20 | *mcr-*7 | *mcr-*7 forward | GCC CTT CTT TTC GTT GTT | |  |  | 551 | |  |  |
|  |  | *mcr*-7 reverse | GGT TGG TCT CTT TCT CGT | |  |  |  | |  |  |
| 21 | *mcr-*8 | *mcr-*8 forward | TCA ACA ATT CTA CAA AGC GTG | |  |  | 856 | |  |  |
|  |  | *mcr*-8 reverse | AAT GCT GCG CGA ATG AAG | |  |  |  | |  |  |
| 22 | *mcr-*9 | *mcr-*9 forward | TTC CCT TTG TTC TGG TTG | |  |  | 1011 | |  |  |
|  |  | *mcr*-9 reverse | GCA GGT AAT AAG TCG GTC | |  |  |  | |  |  |

**Table S2.** The relative expression level of acrB and ramA in 29 efflux pump activity positive tigecycline-resistant C-C-RKP isolate

| **No.** | **Project No.** | **Efflux pump inhibition assay** | | | **Expression level of AcrAB efflux pump** | |
| --- | --- | --- | --- | --- | --- | --- |
|  |  | **TGC MIC** | **TGC MIC + CCCP** | **fold reduction** | ***acrB* regulation** | ***ramA* regulation** |
| 1 | XDR-KP-034 | 1 | 0.25 | 4 | 2.63116 ± 0.112 | 3.96105 ± 0.384 |
| 2 | XDR-KP-077 | 1 | 0.25 | 4 | 1.9035 ± 0.022 | 3.06602 ± 0.158 |
| 3 | XDR-KP-078 | 1 | 0.25 | 4 | 1.24093 ± 0.3 | 2.39167 ± 0.274 |
| 4 | XDR-KP-079 | 1 | 0.25 | 4 | 1.18517 ± 0.09 | 3.22753 ± 0.276 |
| 5 | XDR-KP-080 | 1 | 0.25 | 4 | 1.23635 ± 0.081 | 2.94385 ± 0.619 |
| 6 | XDR-KP-081 | 1 | 0.25 | 4 | 1.19438 ± 0.103 | 3.42677 ± 0.311 |
| 7 | XDR-KP-085 | 1 | 0.125 | 8 | 1.27734 ± 0.331 | 6.26508 ± 0.229 |
| 8 | XDR-KP-091 | 1 | 0.25 | 4 | 1.05115 ± 0.257 | 5.11663 ± 0.255 |
| 9 | XDR-KP-100 | 1 | 0.25 | 4 | 1.36092 ± 0.113 | 4.43585 ± 0.419 |
| 10 | XDR-KP-125 | 1 | 0.25 | 4 | 1.01228 ± 0.169 | 2.7782 ± 0.275 |
| 11 | XDR-KP-173 | 1 | 0.125 | 8 | 1.42365 ± 0.175 | 2.58507 ± 0.153 |
| 12 | XDR-KP-059 | 2 | 0.125 | 16 | 1.50286 ± 0.130 | 1.50367 ± 0.385 |
| 13 | XDR-KP-082 | 2 | 0.5 | 4 | 1.50032 ± 0.05 | 2.97661 ± 0.193 |
| 14 | XDR-KP-083 | 2 | 0.5 | 4 | 1.21129 ± 0.509 | 4.01326 ± 0.556 |
| 15 | XDR-KP-110 | 2 | 0.5 | 4 | 2.49398 ± 0.131 | 9.83044 ± 0.169 |
| 16 | XDR-KP-117 | 2 | 0.5 | 4 | 1.97229 ± 0.129 | 1.93658 ± 0.128 |
| 17 | XDR-KP-189 | 2 | 0.25 | 8 | 1.26462 ± 0.170 | 3.56057 ± 0.251 |
| 18 | XDR-KP-051 | 4 | 1 | 4 | 5.6132 ± 0.178 | 6.31316 ± 0.193 |
| 19 | XDR-KP-069 | 4 | 1 | 4 | 5.79255 ± 0.082 | 24.96536 ± 0.136 |
| 20 | XDR-KP-072 | 4 | 0.125 | 32 | 1.56317 ± 0.173 | 2.45548 ± 0.284 |
| 21 | XDR-KP-158 | 4 | 1 | 4 | 2.12208 ± 0.128 | 2.26396 ± 0.201 |
| 22 | XDR-KP-178 | 4 | 1 | 4 | 1.70586 ± 0.173 | 7.66086 ± 0.326 |
| 23 | XDR-KP-185 | 4 | 1 | 4 | 1.28079 ± 0.326 | 2.06299 ± 0.344 |
| 24 | XDR-KP-187 | 4 | 1 | 4 | 1.75762 ± 0.227 | 1.94432 ± 0.212 |
| 25 | XDR-KP-206 | 4 | 1 | 4 | 4.78159 ± 0.039 | 2.82454 ± 0.338 |
| 26 | XDR-KP-011 | 8 | 1 | 8 | 3.29027 ± 0.265 | 5.0819 ± 0.337 |
| 27 | XDR-KP-012 | 8 | 1 | 8 | 3.01929 ± 0.338 | 6.63876 ± 0.239 |
| 28 | XDR-KP-047 | 8 | 1 | 8 | 4.81836 ± 0.302 | 10.07185 ± 0.043 |
| 29 | XDR-KP-113 | 8 | 1 | 8 | 8.15028 ± 0.364 | 4.32626 ± 0.222 |
